# The Genetic Architecture of Quantitative Traits Cannot Be Inferred from Variance Component Analysis

**DOI:** 10.1371/journal.pgen.1006421

**Published:** 2016-11-03

**Authors:** Wen Huang, Trudy F. C. Mackay

**Affiliations:** 1 Program in Genetics, North Carolina State University, Raleigh, North Carolina, United States of America; 2 W.M.-Keck Center for Behavioral Biology, North Carolina State University, Raleigh, North Carolina, United States of America; 3 Initiative for Biological Complexity, North Carolina State University, Raleigh, North Carolina, United States of America; 4 Department of Biological Sciences, North Carolina State University, Raleigh, North Carolina, United States of America; Case Western Reserve University, UNITED STATES

## Abstract

Classical quantitative genetic analyses estimate additive and non-additive genetic and environmental components of variance from phenotypes of related individuals without knowing the identities of quantitative trait loci (QTLs). Many studies have found a large proportion of quantitative trait variation can be attributed to the additive genetic variance (*V*_*A*_), providing the basis for claims that non-additive gene actions are unimportant. In this study, we show that arbitrarily defined parameterizations of genetic effects seemingly consistent with non-additive gene actions can also capture the majority of genetic variation. This reveals a logical flaw in using the relative magnitudes of variance components to indicate the relative importance of additive and non-additive gene actions. We discuss the implications and propose that variance component analyses should not be used to infer the genetic architecture of quantitative traits.

## Author Summary

There has been a great amount of debate over the relative importance of additivity and non-additivity in quantitative trait variation. The main argument supporting the importance of additivity is the observation that the additive component of genetic variance is much greater than non-additive variance components, while the main argument supporting the importance of non-additivity is the identification of many non-additive effects in genetic mapping studies. By recapitulating many classical results and introducing new alternative parameterizations of genetic effects, we point out some of the common mistakes and misleading arguments in using variance component analyses to infer genetic architecture, specifically the gene actions of QTLs. Because of the wide applications of variance component analyses, our study has profound implications and clarifies some of the most confusing concepts in quantitative genetics in the genomics era.

## Introduction

Nearly a century ago, R. A. Fisher solved the apparent discrepancy between rules of Mendelian inheritance for alleles with large effects at one or a few loci and the resemblance among relatives for quantitative traits with a continuous distribution of phenotypes in natural populations [1,2]. He did this by postulating that many loci with small individual allelic effects caused genetic variation for quantitative traits, and that simultaneously random environmental variation contributed to the continuous phenotypic variation. Rather than assuming the dominant/recessive gene action common for Mendelian loci, he assumed a more general model of gene action at a single locus that could account for any relationship between homozygous and heterozygous allelic effects and derived expectations of the magnitude of genetic variance contributed by many such loci in an outbred population, and, importantly, the expected correlations between common relatives [1,2]. This laid the foundation for the now classical partitioning of genetic variance for quantitative traits in terms of additive (*V*_*A*_), dominance (*V*_*D*_), and inter-locus interaction (epistatic) variance (*V*_*I*_) components [2]. This theory has been exceedingly influential in animal and plant breeding, evolution, and understanding of human complex traits. The additive genetic variance, *V*_*A*_, is of particular importance because it defines the level of narrow sense heritability (*h*^2^), which in turn determines the fraction of the total variance of a quantitative trait that is transmissible from generation to generation, resemblance between relatives and the rate of short-term response to natural or artificial selection from standing variation [3], without knowing the details of the underlying genes.

For the past 25 years, with the advent of molecular markers, the goal of molecular quantitative genetics has been to define the genetic architecture of quantitative traits by identifying the quantitative trait loci (QTLs) underlying quantitative genetic variation as well as the causal molecular variants. One important aspect of the genetic architecture of quantitative traits is the gene actions of QTLs, whether allelic effects are additive within and across loci, one allele is dominant over another, or the effect of one QTL is dependent on the genotype at another locus. The partitioning of genetic variation into *V*_*A*_, *V*_*D*_, and *V*_*I*_ seems to offer a convenient indication of the gene actions of QTLs. For example, the role of epistasis in the genetic architecture of quantitative traits has been surprisingly contentious, despite ample evidence for epistatic interactions between mutations and between quantitative trait loci from studies in model organisms ([4] and references therein) and our general understanding of non-linearity in biochemical, developmental and metabolic networks [5]. The prevailing argument has been that epistasis is not important because it gives rise to mostly *V*_*A*_, and it is *V*_*A*_ that determines correlations among relatives and response to selection [6,7]. Here, we show how this argument arises and why it is misleading; illustrate this point by developing alternative parameterizations of genetic variance that also lead to large proportions of genetic variance apparently due to non-additive gene action; and discuss the implications of the lack of correspondence between homozygous, heterozygous and epistatic interaction effects and additive, dominance and interaction variance components.

## Results

### *V*_*A*_ is a major determinant of total genetic variance under the classical model

**Fig 1:**
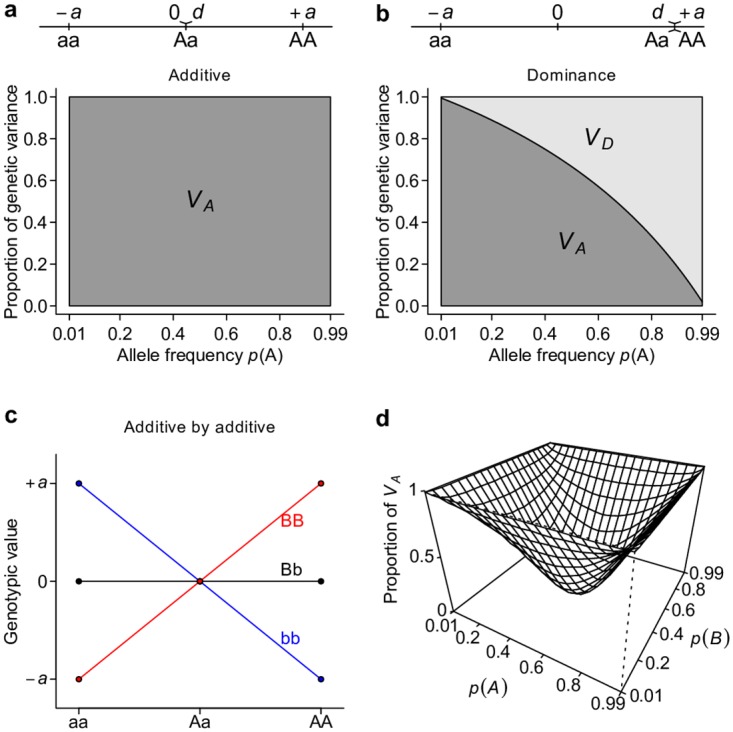
Additive genetic variance *V*_*A*_ is a major determinant of total genetic variance. Under additive (**a**), dominant (**b**), or additive by additive (**c, d**) models, the proportion of total genetic variance explained by the additive genetic variance *V*_*A*_ and dominance genetic variance *V*_*D*_ are estimated either analytically (**a, b**) or numerically by simulation (**d**).

To show the relationship between gene action and classical partitioning of genetic variation, we first consider simple models of genetic architecture that involve one or two loci. Following conventional notation, we arbitrarily assign the genotypic value of the three possible genotypes aa, Aa, and AA at a single bi-allelic locus as −*a*, *d*, and +*a* respectively[2]. Additive and dominant gene actions or genetic models have a clear meaning with this parameterization. An “additive” genetic model refers to the situation in which *d* = 0, and hence there is a perfect linear relationship between the genotypic value and the number of copies of A alleles. A “dominant” genetic model is when *d* = ±*a*, or when the genotypic value is solely determined by the presence of the dominant allele. *V*_*A*_ (see Table 1 for this and other notations and definitions used throughout this study) accounts for the entirety of genetic variation when the true genetic model is an additive model (Fig 1a). *V*_*A*_ also explains the majority of genetic variation under the dominant genetic model unless the dominant allele is at high frequency (Fig 1b). Extending this single-locus model to two unlinked loci, we see that *V*_*A*_ also captures the majority of overall genetic variance—unless alleles at both loci are common—under a two-locus “additive by additive” genetic model (Fig 1c and 1d).

**Table 1:** Notations and definitions of variance components in this study.

Notation	Variance component	Genotype coding
*V*_*A*_	2*pq*[*a* + *d*(*q* − *p*)]^2^	*x*_*A*_*∈* {0, 1, 2}
*V*_*D*_	(2*pqd*)^2^	*x*_*D*_*∈* {0, 2*p*, 2(*p* − *q*)}
$V_{D}^{\prime }$	$\frac{4pq^{2}}{1+q}{\left(a+dq\right)}^{2}$	$x_{D}^{\prime }\in \left\{0,\;2,\;2\right\}$
$V_{A}^{\prime }$	$\frac{2p^{2}q}{1+q}{\left(a-d\right)}^{2}$	$x_{A}^{\prime }\in \left\{0,\;\frac{1-q}{1+q},\;\frac{-2q}{1+q}\right\}$
$V_{AA}^{\prime \prime }$	computed numerically	$x_{AA}^{\prime \prime }\in \left(x_{A,1}-1\right)\left(x_{A,2}-1\right)$

**Fig 2:**
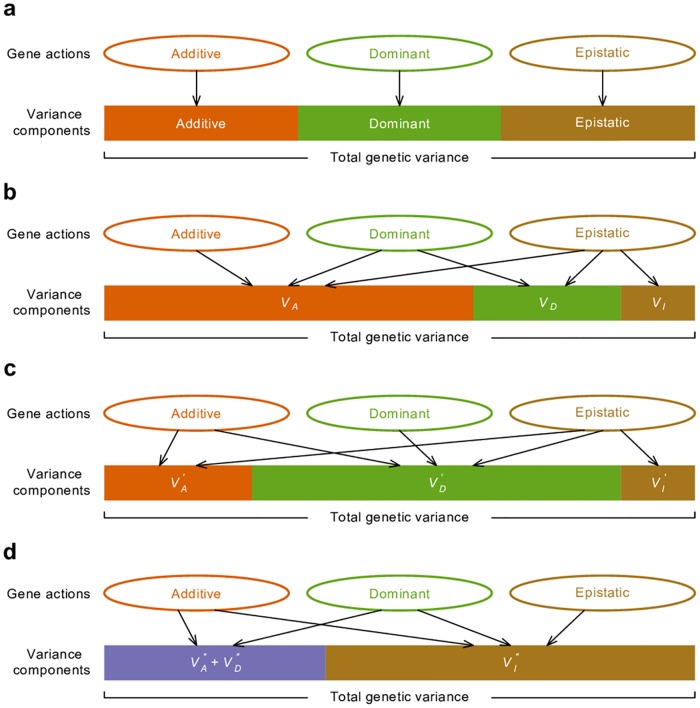
Relationship between gene actions and variance components. (**a**) Ideally, the variance generated by each type of gene actions is mutually exclusive therefore variance components provide a measure of relative importance of gene actions. (**b**) In the classical *V*_*A*_ + *V*_*D*_ + *V*_*I*_ variance partition, additive genetic variance *V*_*A*_ has contribution from all of additive, dominant, and epistatic gene actions in most circumstances. With the alternative parameterizations, all types of gene actions contribute to $V_{D}^{\prime }$ (**c**) and $V_{AA}^{\prime \prime }$ (**d**) in most circumstances.

Ideally, a variance component partition should have a one-to-one corresponding relationship with gene actions in order for it to measure the relative importance of gene actions (Fig 2a). The classical *V*_*A*_ + *V*_*D*_ + *V*_*I*_ partition obviously does not possess this property, despite it being an orthogonal partition (uncorrelated variance components) and having suggestive names, *i*.*e*., additive genetic variance for *V*_*A*,_ dominance genetic variance for *V*_*D*_, and epistatic genetic variance for *V*_*I*_ (Fig 2b). Notably, except for additive gene actions, which contribute only to *V*_*A*,_ both dominant and epistatic gene actions contribute to multiple variance components (Figs 1 and 2b). The specific amount of genetic variation each type of gene action contributes depends on the genetic architecture or may even be unmeasurable because different types of gene actions may not be independent from each other. Nonetheless, it is clear that this classical *V*_*A*_ + *V*_*D*_ + *V*_*I*_ partition is a poor indicator of the underlying genetic architecture; purely epistatic genetic architecture can often result in a partition where *V*_*A*_ is large but *V*_*I*_ is small (Fig 1d). These results are not new and have been previously shown by many authors [2,4,6] but are recapitulated here to set the stage for the following results so that one can contrast alternative parameterizations with them.

The apparent disconnect between gene action and variance components in the classical model is the basis for the statements that epistatic variance *V*_*I*_ can be neglected because epistasis contributes mostly to *V*_*A*_ and *V*_*I*_ is correspondingly small [6]. This is undoubtedly true but vastly misleading. *V*_*I*_ is the residual genetic variance after *V*_*A*_ has been maximized and bears no genetic meaning even though it is called epistatic variance. Indeed, textbooks point out a possible misunderstanding and warn that “*the concept of additive variance does not carry with it the assumption of additive gene action; and the existence of additive variance is not an indication that any of the genes act additively (i*.*e*., *show neither dominance nor epistasis)*” [2].

### Alternative parameterizations also capture the majority of genetic variance

**Fig 3:**
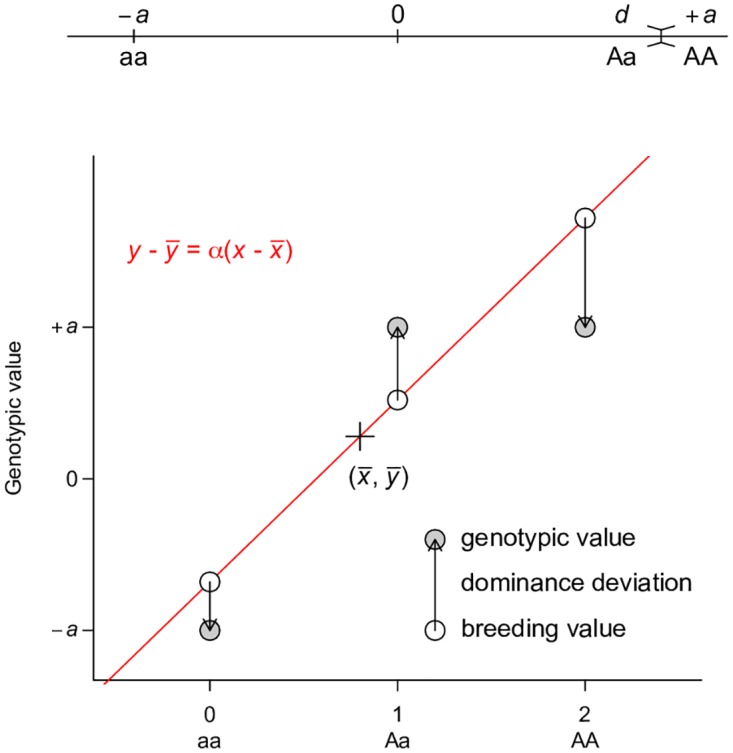
Least squares regression interpretation of *V*_*A*_. This representation is adapted from Fig. 7.2 of Reference [2]. Grey circles indicate the genotypic value of each genotype, which is coded as 0, 1, 2 for aa, Aa, and AA respectively. A regression line (red line) is fitted to the data, on which the fitted values are indicated by white circles. The fitted line must pass through the center of the data, as indicated by the cross. The fitted values are equivalent to breeding values. The arrows between the breeding values and the genotypic values are the dominance deviations, which are the same as residuals of the regression. Note that the data points are weighted by their frequencies in the population. A dominance model is used so that the dominance deviation can be illustrated.

The way genetic effects are parameterized in the *V*_*A*_ + *V*_*D*_ + *V*_*I*_ partition necessarily leads to large *V*_*A*_. This property is best illustrated by the least squares interpretation in a single locus case (Fig 3), in which *V*_*A*_ is the type I sum of squares of regressing genotypic values onto copy number of alleles while *V*_*D*_ is the residual variance. The least squares solution of this regression attempts to maximize *V*_*A*_ and minimize *V*_*D*_ given the assumed additive genetic model, regardless of the actual genetic architecture. The key point, which is often neglected, is to realize that *V*_*A*_ is large not because of the underlying genetic architecture but of the assumed genetic architecture and its corresponding parameterization. When the assumption and parameterization change, the partitioning of variance components also changes.

**Fig 4:**
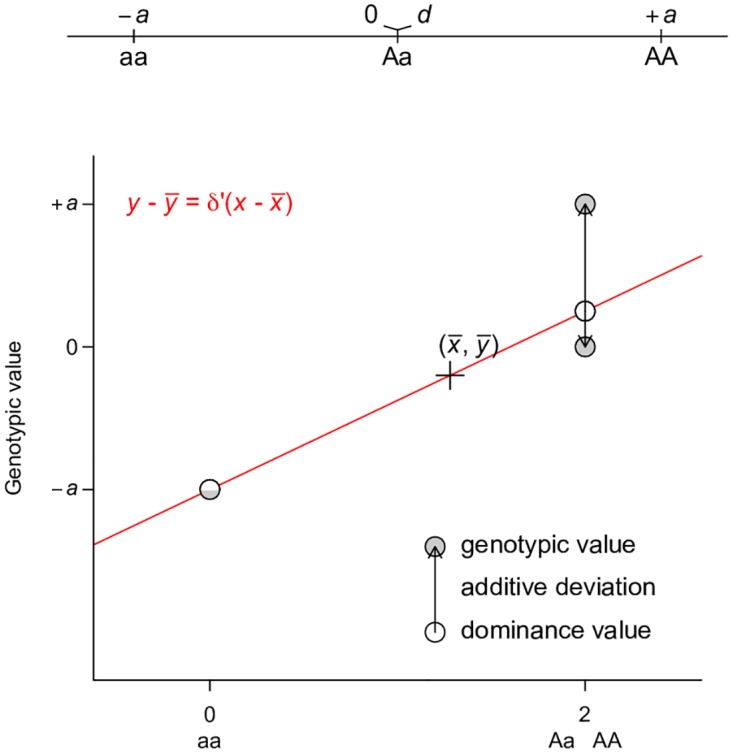
Least squares regression interpretation of $V_{D}^{\prime }$. Grey circles indicate the genotypic value of each genotype, which is coded as 0, 2, 2 for aa, Aa, and AA respectively. A regression line (red line) is fitted to the data, on which the fitted values are indicated by white circles. The fitted line must pass through the center of the data, as indicated by the cross. The fitted line must also pass through the circle (half grey and half white to indicate the overlap of the genotypic and fitted values) denoting genotype aa. The fitted values are equivalent to dominance values as defined in this parameterization. The arrows between the dominance values and the genotypic values are the residuals of the regression, which we define as “additive deviation”, therefore the residual variance is $V_{A}^{\prime }$. Note that the data points are weighted by their frequencies in the population. An additive model is used so that the additive deviation can be illustrated.

**Fig 5:**
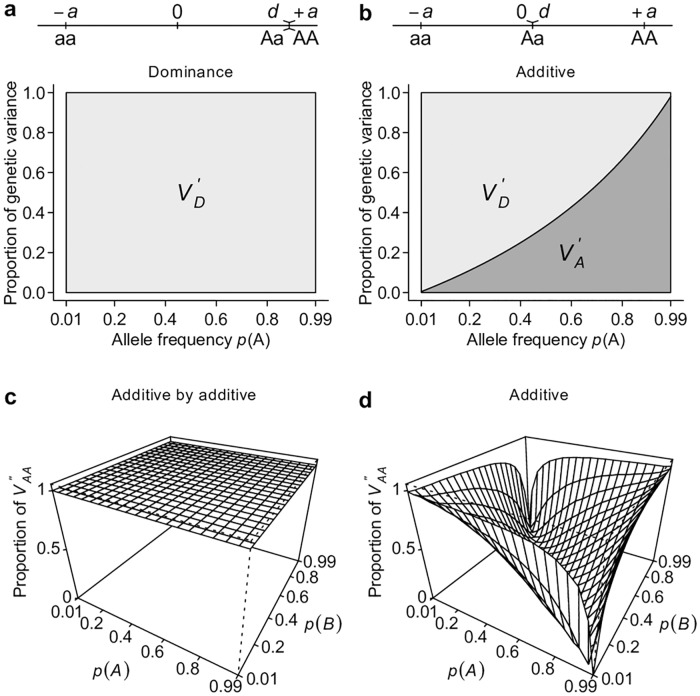
Alternative parameterizations capture the majority of genetic variance. Using an alternative parameterization that emphasizes dominant gene action, a newly defined dominance variance $V_{D}^{\prime }$ and additive deviation variance $V_{A}^{\prime }$ are estimated analytically under dominant (**a**) and additive (**b**) models. Using an alternative parameterization that emphasizes additive by additive gene action, a newly defined interaction variance $V_{AA}^{\prime \prime }$ is estimated numerically under additive by additive (**c**) and additive (**d**) models.

Perhaps the best way to counter the argument that large *V*_*A*_ is evidence for unimportance of non-additive gene actions is to derive alternative ways of partitioning variance where one of the non-additive components dominates others, a property that has been shown previously only for *V*_*A*_. This turns out to be easy if the non-additive components are given the priority to explain the genetic variation, as does *V*_*A*_ in the classical model. Using a single-locus parameterization in which the heterozygotes and the homozygotes for the dominant allele are coded identically, we define an alternative dominance variance $V_{D}^{\prime }$ (Table 1, the prime symbol is used to distinguish this variance from the conventional dominance variance *V*_*D*_), which is the type I sum of squares of regressing genotypic values onto the dominant allelic coding (Table 1; Fig 4). Consistent with its assumed genetic model, $V_{D}^{\prime }$ captures the entire genetic variance when the true genetic model is a completely dominant model (Fig 5a). Even when the genetic model is perfectly additive, $V_{D}^{\prime }$ captures the majority of genetic variation (Fig 5b). This result is remarkable because a variance component $V_{D}^{\prime }$ under the alternative parameterization seemingly corresponding to the dominant gene action has similar properties and variance explaining abilities as *V*_*A*_ (Fig 2c). Furthermore, an alternative two-locus parameterization (see Methods) allows the $V_{AA}^{\prime \prime }$ variance component (Table 1) to explain the entire genetic variance with an additive by additive genetic model (Fig 5c) while still capturing a majority of genetic variance under most circumstances when the genetic model is purely additive (Figs 5d and 2d).

### Classical and alternative parameterizations capture the majority of polygenic genetic variance

**Fig 6:**
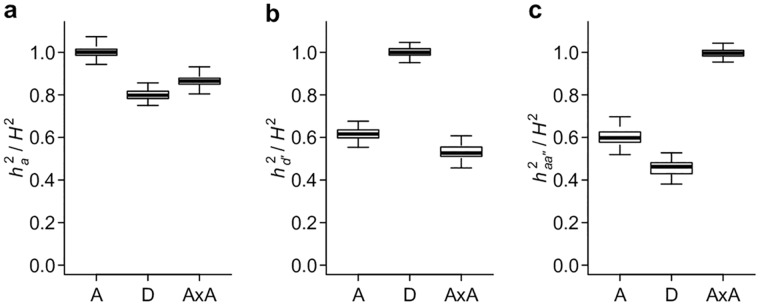
Conventional and alternative parameterizations capture the majority of polygenic genetic variance. Simulation is used to generate data sets with the additive (A), dominant (D), and additive by additive (AxA) genetic models and *V*_*A*,_ $V_{D}^{\prime }$ and $V_{AA}^{\prime \prime }$ are estimated using linear mixed models. The results are presented as the proportion of heritability explained by the genetic variance component; $h_{a}^{2}$ corresponds to *V*_*A*_ (**a**), $h_{d\prime }^{2}$ to $V_{D}^{\prime }$ (**b**), and $h_{aa\prime \prime }^{2}$ to $V_{AA}^{\prime \prime }$ (**c**).

To extend the single- and two-locus results to polygenic genetic models, we simulated genotypes and phenotypes based on pre-defined genetic architectures (gene actions) and broad sense heritability (*H*^2^), and used mixed models to partition phenotypic variance under the classical and alternative parameterizations described above. As expected, when the genetic parameterizations and the corresponding genetic covariance matrices match the true genetic models, the estimated variances fully explain the total genetic variances (Fig 6a–6c). Intriguingly, similar to the single- and two-locus models, all genetic parameterizations are able to capture a large (almost always > 40%) fraction of total genetic variances regardless of the true genetic architecture (Fig 6a–6c). Among the three parameterizations, the classical definition of *V*_*A*_ appears to explain the most genetic variance when the genetic model does not match its parameterization. This is likely because the genotypic coding under the conventional additive parameterization is insensitive to the sign of the allelic effects; while the dominance parameterization requires prior knowledge of the dominant allele, and the additive by additive parameterization requires prior knowledge of the interacting pairs. Nonetheless, it is remarkable that even with random assignment of the dominant allele or random pairing of loci and obvious mischaracterization of the genetic model, $V_{D}^{\prime }$ and $V_{AA}^{\prime \prime }$ are able to explain the majority of genetic variance when the genetic architecture is additive within and between loci, respectively (Fig 6b and 6c).

### *V*_*A*_, $V_{D}^{\prime }$, and $V_{AA}^{\prime \prime }$ explain a large fraction of phenotypic variance for human height

**Fig 7:**
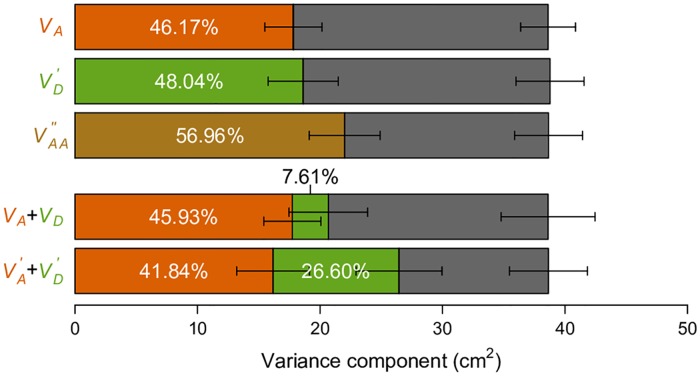
Variance component analyses of human height data. Phenotypic variation of height (in cm) observed in the GENEVA study is partitioned into genetic variance components as indicated (color-coded bars) and environmental variance (*V*_*e*_, grey bar). The colors of bars correspond to the colors of the text indicating the variance components. Error bars indicate standard errors of the variance component estimates provided by GCTA. Proportions of the components are also indicated.

It has been previously shown that *V*_*A*_ accounts for a large fraction of phenotypic variance in human adult height using a genetic covariance matrix computed from genome-wide SNP data under the conventional parameterization [8]. Based on our above results, we necessarily expect this observation regardless of the true genetic architecture for human height. We indeed recapitulated this result using genotype and height data for individuals from the GENEVA project (Fig 7). We then asked if our alternative parameterizations of $V_{D}^{\prime }$ and $V_{AA}^{\prime \prime }$ can perform with real data as they do in simulated data (Fig 6b and 6c). Remarkably, under the naive assumptions that minor alleles are recessive and by randomly pairing interacting SNPs, both $V_{D}^{\prime }$ and $V_{AA}^{\prime \prime }$ can explain a substantial fraction of phenotypic variance, with even larger point estimates than *V*_*A*_ (Fig 7). This is important, because $V_{D}^{\prime }$ and $V_{AA}^{\prime \prime }$ and their corresponding parameterizations are consistent with dominant and epistatic gene actions. If we use the same argument that large *V*_*A*_ in the classical model suggests the unimportance of non-additive gene actions, large $V_{D}^{\prime }$ and $V_{AA}^{\prime \prime }$ in the alternative parameterizations suggest the unimportance of additive gene actions. Neither argument is correct.

To further illustrate how parameterizations may affect genetic variance partitioning, we focus on the simple task of partitioning “additive” and “dominance” variances. A recent study reported a major contribution of *V*_*A*_ (additive variance) and a minor contribution of *V*_*D*_ (dominance variance) for a number of quantitative traits, using the classical parameterization for the additive genetic variance to estimate *V*_*A*,_ and a frequency-dependent parameterization (Table 1) orthogonal to the additive genetic value to estimate *V*_*D*_[9]. This observation led to the conclusion that dominance variation contributes little to quantitative trait variation. We observed similar relative contributions of *V*_*A*_ and *V*_*D*_ for human height in the GENEVA data (Fig 7). However, as we have illustrated above, this is only one of the many possible ways of partitioning variance. Using our alternatively defined parameterizations and a similar frequency-dependent parameterization orthogonal to the dominant genetic value (Table 1), we find a much more substantial contribution of dominance variance, *i*.*e*., $V_{D}^{\prime }$ (Fig 7). The key difference between this alternative partition $V_{D}^{\prime }+V_{A}^{\prime }$ and the classical *V*_*A*_ + *V*_*D*_ (*V*_*I*_ is ignored here) is that the variance component seemingly consistent with the dominant gene action is allowed to explain the variance first, while the “additive” component ($V_{A}^{\prime }$) only enters the model after $V_{D}^{\prime }$ has been maximized (Fig 4). This result clearly demonstrates the problem of using variance partitioning to measure the relative importance of gene actions, because they change as the parameterizations and models change.

## Discussion

Here, we have re-iterated the well-known observation from classical quantitative genetics theory that *V*_*A*_ contributes the majority of the genetic variance in an outbred population regardless of the underlying gene actions at individual loci and across loci [2,6]. However, the inference that therefore, epistatic gene action can be ignored because the epistatic variance *V*_*I*_ is small is not correct, although this viewpoint is pervasive in the literature. For example, Crow [7] concludes that epistasis is unimportant in polygenic selection on the basis that most genetic variation is *V*_*A*,_ ignoring the fact that epistasis contributes to *V*_*A*_. Bloom *et al*. [10] use the observation that the small difference between *H*^2^ (which measures total genetic variance) and *h*^2^ (which measures additive genetic variance) for some yeast quantitative traits as evidence that there is little epistasis, mistakenly equating *V*_*A*_ with variance due only to additive gene actions. Maki-Tanila and Hill (2015) analytically derive multi-locus models showing that “*epistasis makes substantial contributions to additive variance*”, while at the same time inferring that the existence of epistasis “*does not imply that … it will contribute much genetic variation*” [11]; an apparent inconsistency since it contributes substantially to *V*_*A*._

We have shown that our alternative parameterizations for $V_{D}^{\prime }$ and $V_{AA}^{\prime \prime }$ also capture the majority of the total genetic variance in simulated as well as real data. Using the same incorrect logic, we could infer that $V_{A}^{\prime }$ and $V_{I}^{\prime }$ are unimportant using the $V_{D}^{\prime }$ parameterization; and $V_{A}^{\prime \prime }$ and $V_{D}^{\prime \prime }$ are unimportant using the $V_{AA}^{\prime \prime }$ parameterization. Neither the classical nor the alternative partitions of genetic variance offers any information regarding whether the majority of variance is due to a specific type of gene action. In natural populations, whether one of these ways of variance partitioning is more useful than another depends on the true underlying genetic models. Though it is impossible to determine a clear winner, it is obvious that *V*_*A*_ fully explains genetic variation under an additive model, $V_{D}^{\prime }$ under a dominant model, and $V_{AA}^{\prime \prime }$ under an additive by additive epistatic model. Therefore it seems only appropriate to define *V*_*A*_ as the additive variance when the genetic model is additive, but to define $V_{D}^{\prime }$ rather than *V*_*D*_ as the dominance variance when the genetic model is dominant, and to define $V_{AA}^{\prime \prime }$ as the epistatic variance when the genetic model is entirely additive by additive.

The crux of the problem is the undesirable feature of the classical model as well as the alternative parameterizations that there is not a one-to-one correspondence between gene action at underlying quantitative trait loci and the partitioning of variance components except under very specific and restrictive circumstances (Fig 2). Under the classical model, epistasis and dominant gene action both contribute to *V*_*A*,_ so the relative magnitude of different gene actions cannot be inferred from the relative magnitude of different genetic variance components. A large *V*_*A*_ and small *V*_*D*_ and *V*_*I*_ mean nothing more than a specific partition of genetic variance and there are potentially an infinite number of such partitions, some having larger seemingly additive components than others.

The ability of arbitrarily defined parameterizations to capture the majority of genetic variance shares analogy with the ability of the type I sum of squares to explain variance that is not always attributable to the experimental factor when the experimental design is not orthogonal. In genetic studies, an orthogonal design is not always achievable and impossible in natural populations. In fact, partitioning genetic variance according to different gene actions can be thought of as defining experimental treatments after the experiments have been performed. This is clearly not an ideal statistical practice, though there is no obvious alternative. There have been many attempts to partition genetic variance with the aim to better reflect the underlying contribution of different types of gene actions [12–14]. All of these methods involve certain ways of coding genotypes to partition genetic effects. To ensure that the variance components are uncorrelated or orthogonal, these genotyping codings typically need to be allele frequency dependent. Despite these clever attempts and useful properties in some circumstances, their usefulness is limited because in most cases, any attempt to partition variance into components that correspond to different types of gene actions is destined to fail unless the gene actions happen to be statistically orthogonal. However, the additive and dominant gene actions as commonly defined (Fig 1a and 1b) are two intrinsically inseparable terms and not orthogonal. For an allele to be dominant over another (*a* ≠ 0,*d* = ±*a*), there must necessarily be additive homozygous effects (*a* ≠ 0). This is the root of the confusing convolution of different variance components, especially when not clearly defined.

Although it is not informative about genetic architecture and has its own problem when the genetic architecture does not fit the assumptions [15], we are not suggesting that the classical method of partitioning variance components be abandoned. The classical model, and particularly the concepts of *V*_*A*_ and *h*^2^, have been and will continue to be the foundation of quantitative genetics, predicting resemblance between relatives and response to selection: they inform us about the proportion of phenotypic variation that is “breedable” [3]. The brilliance of this model is that it describes the behavior of quantitative traits across generations in the absence of detailed knowledge of the elements of the genetic system. We are also not suggesting that our alternative parameterizations are in the least bit useful—they are not. We constructed them as illustrative examples of the fallacy of the argument proposing that a mode of gene action is not important because the first variance component fit in the model subsumes contributions from that model of gene action. Rather, we are suggesting that we understand and accept the limitations of the assumptions of the classical model and do not relate empirically useful parameters such as *V*_*A*_ and *h*^2^ to any inferences of underlying gene action. Furthermore, rapid conceptual and technological advances are presenting new challenges, therefore the classical paradigm needs expansion, modification, or revolution to cope with these challenges [16].

We need to separate the goal of using quantitative genetics to predict phenotypes across generations with that of understanding the molecular genetic architecture of complex traits and predicting individual quantitative trait phenotypes from genotypes, which is a within-generation endeavor. This is especially true for understanding and utilizing non-additive gene actions because contributions to phenotypes from dominance and epistasis are not transmissable to the next generation. For example, under an epistatic model in which a particular combination of alleles at multiple loci causes disease, we want to know the susceptible genotype to predict individual phenotype as well as the molecular mechanisms of the genetic interaction, which requires knowledge of the genetic model. This is akin to the well-known distinction between “statistical” and “physiological” epistasis, where the former is concerned with variance component decomposition and the latter with genes and gene action [12,14]. This distinction is not possible when QTLs are unknown, such is the case in classical biometrical treatment of quantitative traits. With the availability of abundant molecular markers, QTLs can be mapped with great precision. This provides the basis to determine the gene actions of mapped QTLs rather than using variance component analysis to infer them, which, as we have demonstrated, is impossible. Determination of genetic architecture even after QTLs are identified is non-trivial and may involve a combination of statistically evaluating and experimentally testing different models, such as through editing specific genes in a defined genetic background. Nonetheless, the controversy over the importance of epistasis can only be resolved by mapping all QTLs and determining their modes of inheritance.

## Methods

### Least squares regression interpretation of *V*_*A*_

Consider a single biallelic locus in a diploid genome with alleles A and a, each with frequency *p* and *q*(*p* + *q* = 1); and assign genotypic values *y* = − *a*, *d*, and +*a* to genotypes aa, Aa, and AA respectively. The average effects of A and a are then *qα* and − *pα* respectively, where *α* = *a* + *d*(*q* − *p*) is the allele substitution effect and measures the change in phenotype in an individual if an allele a is substituted with A [2]. The breeding value, defined as the expected genotypic value of the progeny an individual produces, is the sum of average allelic effects each diploid individual carries, and is − *2pα*, *qα* − *pα*, and *2pα* for aa, Aa, and AA respectively. With only one locus, the total genetic variation in a randomly mating (thus in Hardy-Weinberg equilibrium) population can be partitioned into two orthogonal components, the additive genetic variance *V*_*A*_, which is defined as the variance due to breeding values, 2*pqα*^2^, and the dominance genetic variance *V*_*D*_ = (2*pqd*)^2^ (Table 1) [2].

Alternatively, we can define a random variable *x*_*A*_ as:

$$x_{A}\;=\{\begin{array}{cc} 0, & genotype=aa,\\ 1, & genotype=Aa,\\ 2, & genotype=AA. \end{array}$$

This parameterization has the convenient interpretation that *x*_*A*_ is equal to the number of A alleles. It is easy to show that the allele substitution effect *α* as defined above is the slope of the least squares regression of genotypic value *y* on *x*_*A*_ in an idealized population with random mating (Fig 3). The additive genetic variance is then *V*_*A*_ = *Var*(*ŷ*) = *Var*(*αx*_*A*_) = α^2^*Var*(*x*_*A*_) = 2*pqα*^2^ and the dominance genetic variance *V*_*D*_ is the residual variance. It is easy to see that the least squares solution for this regression seeks to maximize *V*_*A*_ and minimize *V*_*D*_. This least squares interpretation is not new and dates back to the early days of quantitative genetics [1].

By extension of this least squares regression interpretation of genetic variation, if we arbitrarily define any one random variable *x* or more than one of them and fit a linear model of form *y* = *βx* + *ϵ*, we can partition genetic variance due to the assumed genetic model *Var*(*ŷ*) = *Var*(*βx*) and residual variance *Var*(*ϵ*).

### Derivation of dominance variance $V_{D}^{\prime }$ using least squares regression

Now we illustrate the idea of using least squares regression to partition genetic variance due to dominant gene action and the remaining genetic variance. We define the random variable $x_{D}^{\prime }$ as:

$$x_{D}^{\prime }=\{\begin{array}{cc} 0, & genotype=aa;\\ 2, & genotype=Aa\;or\;AA. \end{array}$$

The least square solution for the linear model $y=\delta \prime x_{D}^{\prime }+\epsilon$ can be easily found to be $\delta^{\prime }=\frac{q}{1+q}d+\frac{1}{1+q}a$ (Fig 4). Therefore the variance due to $x_{D}^{\prime }$ is $V_{D}^{\prime }=\frac{4pq^{2}}{1+q}{\left(a+dq\right)}^{2}$. The residuals from this regression are 0, $\frac{1-q}{1+q}\left(d-a\right)$, and $\frac{-2q}{1+q}\left(d-a\right)$, for genotypes aa, Aa, and AA respectively. Similar to *V*_*A*_ and *V*_*D*_, we define the residual variance as an “additive deviation” variance $V_{A}^{\prime }$, which can be found to be $V_{A}^{\prime }=\frac{2p^{2}q}{1+q}{\left(a-d\right)}^{2}$.

### Finding $V_{AA}^{\prime \prime }$ numerically

Extending the least squares regression interpretation of genetic variance to any arbitrary random variable *x* and finding the solution is not always easy. However, it is computationally trivial to find. For example, to numerically estimate the additive by additive variance $V_{AA}^{\prime \prime }$, we define $x_{AA}^{\prime \prime }$ as follows for two independently segregating loci with alleles A/a, and B/b respectively:

$$x_{1}=\{\begin{array}{c} -1,\;\;genotype=aa;\\ 0,\;\;genotype=Aa;\\ 1,\;\;genotype=AA. \end{array},\;x_{2}=\{\begin{array}{c} -1,\;\;genotype=bb;\\ 0,\;\;genotype=Bb;\\ 1,\;\;genotype=BB. \end{array}$$

Then, $x_{AA}^{\prime \prime }=x_{1}x_{2}$. We randomly draw 100,000 individuals with the specific genotypes according to pre-defined allele frequencies and assign genotypic values with pre-defined genetic models. The slopes $\hat{\beta }$ can be easily found by numerically regressing *y* onto *x*. The proportion of genetic variation explained by this parameterization is then just the *R*^2^ of the regression.

### Mixed model analysis of simulated and real data

To extend the single- and two-locus models to polygenic models, we used mixed model analysis to partition phenotypic variation in simulated and real data. To simulate phenotypic data with pre-defined genetic models, we first drew 1,000 realizations from the U-shaped distribution [6] $f\left(p\right)\propto \frac{1}{pq}$, which took possible values of 0.01, 0.02, …, 0.99. Genotypes for these *p* = 1,000 loci were randomly assigned according to their Hardy-Weinberg frequencies to *n* = 5,000 individuals. Genetic values were then assigned to the 5,000 individuals using this general formula **g** = **Xβ**. Each of the columns of the *n* × *p* matrix **X** was coded by the additive parameterization *x*_*A*_ as defined above for the additive genetic model, $x_{D}^{\prime }$ for the dominance genetic model. Similarly for the additive by additive genetic model, $\frac{p}{2}=500$ pairs of loci were parameterized as defined above using $x_{AA}^{\prime \prime }$. The vector **β** was drawn from standard normal distribution. The phenotypic value *y* for each individual was then simulated by adding random noise such that **Y** = **g** + **ϵ**, where **ϵ** was normally distributed with zero mean and variance equal to $Var\left(g\right)\frac{1-H^{2}}{H^{2}}$. *H*^2^ was the broad sense heritability and was always set to 0.5.

We standardized columns of **X** and computed the covariance matrix as **XX**^**T**^, which was further scaled by the mean of its diagonal values. A linear mixed model **Y** = *μ***1** + **Z*u*** + **ϵ** was fitted to the data, where *μ* was the population mean, **Z** was the incidence matrix and in all cases in this study the identity matrix, ***u*** was a random effect with variance covariance matrix **G**σ^2^, where **G** was simply the scaled **XX**^**T**^ above and σ^2^ was the part of genetic variance due to the specific parameterization. We fitted this model using the GCTA software[17] with REML and performed simulations 100 times. We defined the heritability explained by σ^2^ as *h*^2^/*H*^2^, where $h^{2}=\frac{\sigma^{2}}{\sigma^{2}+\sigma_{\epsilon }^{2}}$, and *H*^2^ was the simulated broad sense heritability.

To analyze real data where the genetic architecture cannot be known *a priori*, we downloaded genotype and phenotype data from dbGaP for the GENEVA Genes and Environment Initiatives in Type 2 Diabetes study (phs000091.v2.p1). We pruned the data set to contain 5,497 unrelated (nominal genetic relationship as calculated by GCTA < 0.05) individuals with European ancestry based on both self-reported ethnicity and principal component analysis. We then computed genetic covariance matrices as defined above using autosomal SNPs and partitioned phenotypic variance using GCTA where sex was fitted as a fixed effect in the model. We used the parameterization (Table 1) as defined in a recent study[9] to partition phenotypic variance into *V*_*A*_, *V*_*D*_, and *V*_*e*_. We also partitioned phenotypic variance into $V_{A}^{\prime }$, $V_{D}^{\prime }$, and *V*_*e*_, where $V_{D}^{\prime }$ was defined as above and $V_{A}^{\prime }$ was estimated by defining a new variable $x_{A}^{\prime }$ (Table 1), where

$$x_{A}^{\prime }=\{\begin{array}{c} 0,\;\;genotype=aa,\\ \frac{1-q}{1+q},\;\;genotype=Aa,\\ \frac{-2q}{1+q},\;\;genotype=AA. \end{array}$$
